# *lin-4* and the NRDE pathway are required to activate a transgenic *lin-4* reporter but not the endogenous *lin-4* locus in *C*. *elegans*

**DOI:** 10.1371/journal.pone.0190766

**Published:** 2018-01-11

**Authors:** Alan L. Jiao, Daniel J. Foster, Julia Dixon, Frank J. Slack

**Affiliations:** 1 Institute for RNA Medicine, Department of Pathology, Beth Israel Deaconess Medical Center, Harvard Medical School, Boston, MA, United States of America; 2 Department of Molecular, Cellular, and Developmental Biology, Yale University, New Haven, CT United States of America; 3 Department of Biological and Biomedical Sciences, Harvard University, Boston, MA, United States of America; National Institutes of Health, UNITED STATES

## Abstract

As the founding member of the microRNA (miRNA) gene family, insights into *lin-4* regulation and function have laid a conceptual foundation for countless miRNA-related studies that followed. We previously showed that a transcriptional *lin-4* reporter in *C*. *elegans* was positively regulated by a *lin-4*-complementary element (LCE), and by *lin-4* itself. In this study, we sought to (1) identify additional factors required for *lin-4* reporter expression, and (2) validate the endogenous relevance of a potential positive autoregulatory mechanism of *lin-4* expression. We report that all four core nuclear RNAi factors (*nrde-1*, *nrde-2*, *nrde-3* and *nrde-4*), positively regulate *lin-4* reporter expression. In contrast, endogenous *lin-4* levels were largely unaffected in *nrde-2;nrde-3* mutants. Further, an endogenous LCE deletion generated by CRISPR-Cas9 revealed that the LCE was also not necessary for the activity of the endogenous *lin-4* promoter. Finally, mutations in mature *lin-4* did not reduce primary *lin-4* transcript levels. Taken together, these data indicate that under growth conditions that reveal effects at the transgenic locus, a direct, positive autoregulatory mechanism of *lin-4* expression does not occur in the context of the endogenous *lin-4* locus.

## Introduction

The *lin-4* miRNA is the founding member of the miRNA gene family, and a critical regulator of developmental timing in *C*. *elegans* [1]. *lin-4* loss-of-function mutants display severe developmental phenotypes, including abnormal seam cell division and differentiation patterns, and a complete failure in vulval morphogenesis [2]. *lin-4* is strongly upregulated toward the end of the first larval (L1) stage, resulting in the suppression of its key target, *lin-14*, to promote L2-specific developmental events [3]. Previous reports have identified the FLYWCH (FLH) family of transcription factors [4], as well as the Period homolog LIN-42 [5–7], as repressors of *lin-4* expression during *C*. *elegans* development. However, FLH transcription factors were found to primarily affect embryonic *lin-4* expression, whereas *lin-4* was only mildly de-repressed in *lin-42* mutant larvae. Thus, during larval development, the molecular mechanisms that regulate the timing and activation of *lin-4* expression remain unclear.

miRNA promoter::GFP gene fusions have been instrumental in uncovering the transcriptional regulation and expression patterns of numerous miRNAs. A ~500bp promoter region is sufficient to drive *lin-4* expression and rescue the *lin-4(e912)* null phenotype [1]. We and others have shown that animals carrying a GFP reporter driven by this ~500bp *lin-4* promoter (*Plin-4*::*GFP)* begin expressing GFP in the seam cells in late L1, consistent with the reported timing of *lin-4* upregulation as measured by Northern blotting and quantitative PCR (qPCR) [8,9]. Moreover, we discovered that a *lin-4* complementary element (LCE), as well as *lin-4* itself, was necessary for *Plin-4*::*GFP* expression [10]. This suggested that the *lin-4* miRNA may function in a highly non-canonical manner to transcriptionally activate its own expression.

miRNAs typically function in the RNA interference (RNAi) pathway, acting as specificity factors to recruit Argonaute proteins to silence targeted transcripts [11]. In contrast, the term RNA activation, or “RNAa”, has been used to describe a phenomenon by which small RNAs complementary to promoter regions induce the transcriptional activation of the downstream gene [12]. While the mechanisms of RNAa remain poorly understood, one mechanistic similarity between RNAa and RNAi appears to be the requirement of an Argonaute protein as the effector of gene regulation [13]. In this study, we report that the four major nuclear RNAi (*nrde*) factors in *C*. *elegans*, including the nuclear Argonaute NRDE-3 (nuclear RNAi defective 3) [14,15], are necessary for *Plin-4*::*GFP* expression. Taken together with our previous work [10], these findings strongly supported the model of a direct, positive feedback loop in the regulation of *lin-4* expression.

However, we further show that this potential autoregulatory mechanism is not active at the endogenous *lin-4* locus under the conditions examined here. *nrde* mutants did not display significantly altered *lin-4* expression, nor any detectable *lin-4* phenotypes. Similarly, CRISPR-Cas9-mediated mutations of the LCE and of the mature *lin-4* sequence did not result in any measurable effects on endogenous *lin-4* promoter activity. Thus, our work describes a gene regulatory mechanism active in a transgenic context, but not at the endogenous gene locus. These results emphasize the importance of validating the endogenous relevance of *cis-*regulatory elements identified through reporter-based experiments.

## Results and discussion

### *lin-4* reporter expression is activated by nuclear RNAi factors

**Fig 1:**
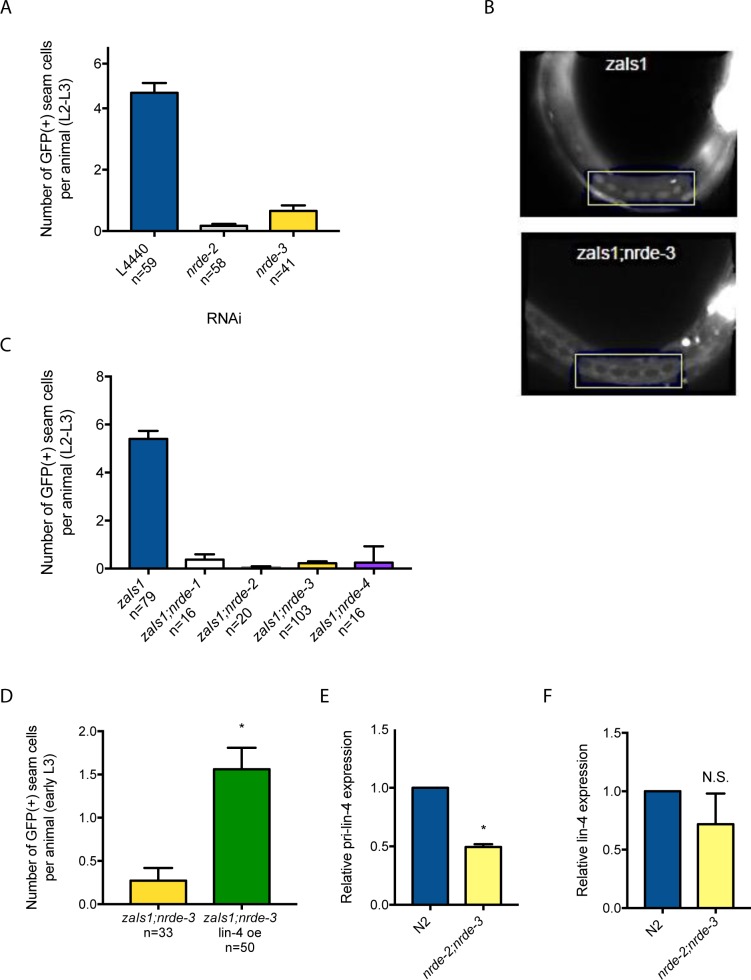
Nuclear RNAi factors activate *lin-4* reporter expression in *C*. *elegans* seam cells. (A) Quantification of GFP-positive seam cells in L2-L3 *zaIs1(Plin-4*::*GFP)* animals grown on indicated RNAi clones. L4440 is the empty vector control. Error bars represent SEM. (B) Fluorescent images of a late L2 *zaIs1* (top) and early L3 *zaIs1*;*nrde-3* (bottom). Rectangle encloses seam cells. (C, D) Quantification of GFP-positive seam cells in L2-L3 animals. *lin-4* oe = *lin-4* overexpression. Error bars represent SEM. (E, F) qPCR analysis of primary and mature *lin-4* expression in synchronized L1’s (at 12h post-embryonic development). Error bars represent SD of at least two independent experiments. * p < 0.05, two-tailed t-test.

The *lin-4* miRNA and an LCE in the *lin-4* promoter are required for the expression of a *Plin-4*::*GFP* reporter [10]. Given that miRNAs function within Argonaute complexes, we hypothesized that *lin-4* may bind to the LCE and activate *Plin-4*::*GFP* expression through the action of the *nrde* genes. To test this, we depleted *nrde-2* and *nrde-3* by RNAi in the *zaIs1(Plin-4*::*GFP)* line, and measured the effect on seam cell GFP expression. Both RNAi treatments significantly reduced seam cell GFP in L2 and L3 animals, suggesting that *nrde-2* and *nrde-3* are positive regulators of *Plin-4*::*GFP* activity (Fig 1A). To validate these results, and to investigate additional *nrde* genes, we crossed the *zaIs1(Plin-4*::*GFP)* line into *nrde-1*, *nrde-2*, *nrde-3* and *nrde-4* mutant animals. We found that seam cell GFP expression was completely abolished in each of the four *nrde* mutant backgrounds (Fig 1B and 1C). We conclude that the nuclear RNAi pathway is required for the expression of a *lin-4* reporter in *C*. *elegans* seam cells.

*lin-4* overexpression is sufficient to upregulate *Plin-4*::*GFP* activity [10]. To test if this was dependent on NRDE-3, we overexpressed *lin-4* in the *zaIs1;nrde-3* mutant line. Similar to what we previously observed in a wild-type background, we found that *lin-4* overexpression resulted in a ~four-fold increase in seam cell GFP expression (Fig 1D). These data suggest that the nuclear Argonaute NRDE-3 is not required for the *lin-4-*mediated activation of *Plin-4*::*GFP* expression. Thus, the precise mechanisms through which both *lin-4* and the *nrde* pathway activate *lin-4* reporter expression remain to be determined. While the nuclear RNAi pathway is well known to silence transgenes [16], our data represents, to our knowledge, the first example of *nrde* factors in positively affecting the expression of a transgene. How this occurs in the context of the *lin-4* reporter may provide new insights into small RNA pathways and the balance between silencing and allowing/inducing the expression of different foreign sequences.

### Nuclear RNAi factors are not required for mature *lin-4* expression

Do the *nrde* genes also positively regulate endogenous *lin-4* expression? To test this, we generated a *nrde-2;nrde-3* double mutant, and performed qPCR to measure primary and mature *lin-4* levels in synchronized (12h) L1’s–a time at which *lin-4* expression begins to increase during wild-type development. We observed only a mild decrease in primary *lin-4* levels in *nrde-2;nrde-3* mutants compared to wild-type, without a significant decrease in mature *lin-4* (Fig 1E and 1F). To determine whether the NRDE pathway may regulate endogenous *lin-4* expression specifically in seam cells, we examined two seam cell phenotypes which manifest with 100% penetrance in *lin-4(e912)* null mutants. First, we examined the L2-specific division in V-lineage seam cells, which fail to occur in the absence of *lin-4*. Second, we examined the formation of adult alae, a cuticular structure that does not form in *lin-4(e912)* mutants due to a failure of seam cell differentiation. We found that the timing of L2 seam cell divisions was unaffected in *nrde-3* mutants, while adult alae formation was also unaffected in *nrde-2;nrde-3* mutants (Table 1). Taken together, these results suggest that while *nrde-2* and *nrde-3* are required for seam cell *Plin-4*::*GFP* activity, they do not regulate endogenous *lin-4* expression.

**Table 1:** L2 seam cell divisions and adult alae are unaffected in *nrde-3* and *nrde-2;nrde-3* mutants, respectively.

% Animals Completed L2-Specific Seam Cell Divisions	
*wIs79*	86% (n = 87)
*wIs79;nrde-3*	85% (n = 105)
% Animals with Adult Alae	
N2	72% (n = 44)
*nrde-2;nrde-3*	70% (n = 54)

The *wIs79(ajm-1*::*GFP; scm-1*::*GFP)* seam cell marker strain was used to visualize seam cell divisions. Synchronized L1’s were plated on op50 and were grown at 25°C for 15h prior to scoring. Animals were scored positive as long as at least one of the six V-lineage seam cell completed the L2-specific symmetrical division prior to a second, asymmetrical division, producing a maximum of six additional seam cells. To examine the presence of alae in young adults, synchronized L1’s were plated on op50 and scored 54h post-feeding.

### Deletion of the endogenous LCE does not affect mature *lin-4* expression

**Fig 2:**
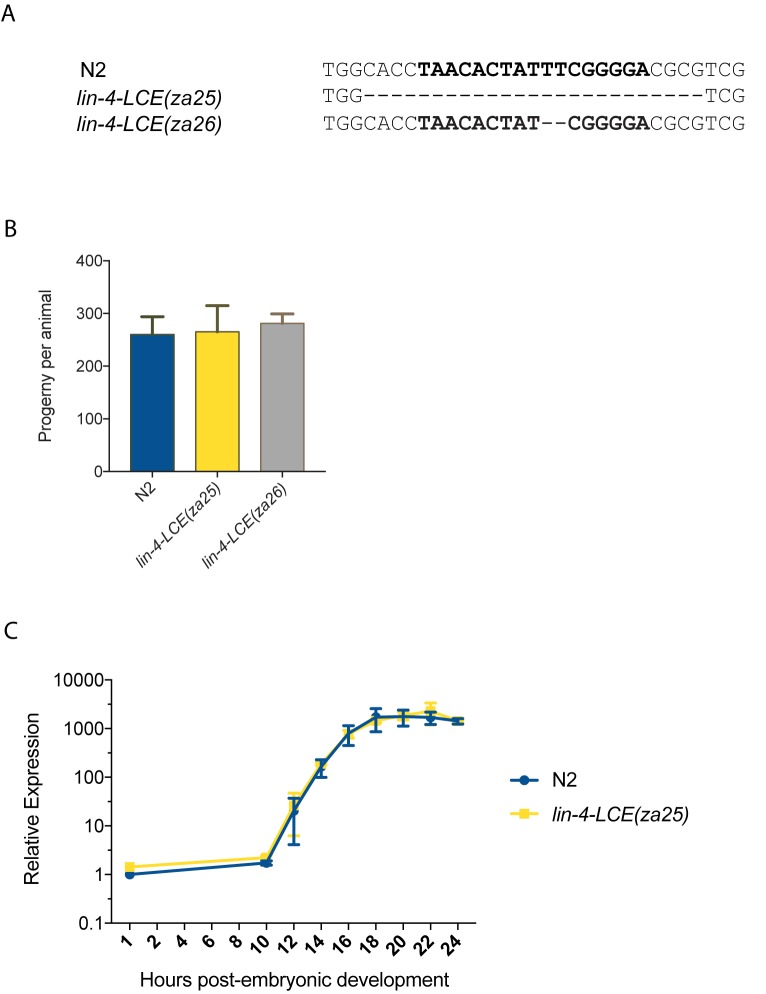
The LCE is not required for mature *lin-4* expression. (A) The sequence of the LCE in wild-type (N2) animals is in bold. Below are the aligned sequences of two CRISPR-Cas9-generated LCE mutants. Dashes indicate deleted bases. (B) Brood size assay of LCE mutants, expressed as a mean ± SD (n >7). (C) qPCR time course of mature *lin-4* expression. Mature *lin-4* expression relative to U18 were all normalized to the 1hr N2 time point, and expressed as a mean ± SD of two independent experiments.

The LCE in the *lin-4* promoter is essential for the expression of a *Plin-4*::*GFP* reporter [10]. To test the regulatory importance of the LCE in the endogenous *lin-4* locus, we targeted the LCE using CRISPR-Cas9 [17] and generated two LCE mutant *C*. *elegans* lines. *lin-4-LCE(za25)* harbors a 25nt deletion that removed the entire 17nt LCE and 4nt on either side; *lin-4-LCE(za26)* harbors a 2 nucleotide (TT) deletion (Fig 2A). These mutants were backcrossed into a wild-type (N2) background three times before further analysis.

Both LCE mutants appeared morphologically wild-type, and displayed no obvious *lin-4* phenotypes, including no significant changes in brood size (Fig 2B). In wild-type animals, we found *lin-4* to be upregulated ~1500-fold between 10h and 16h of post-embryonic development (Fig 2C); however, the timing and magnitude of this upregulation was unaffected in *lin-4-LCE(za25)* mutants. We conclude that deletion of the endogenous LCE does not affect mature *lin-4* miRNA expression.

### The LCE is not required for endogenous *lin-4* promoter activity

**Fig 3:**
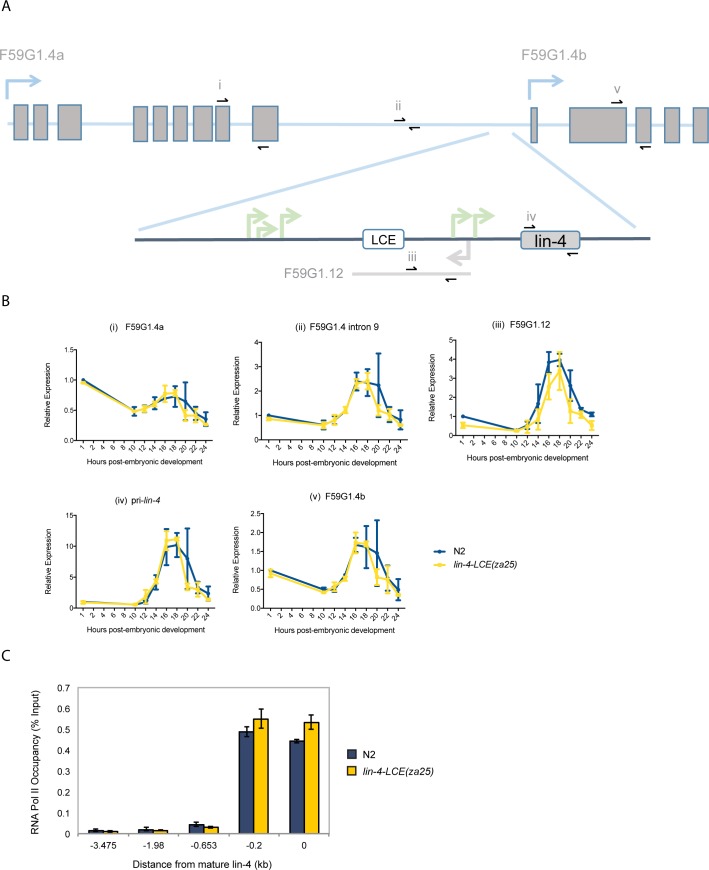
The LCE is not required for endogenous *lin-4* promoter activity. (A) Structure of the F59G1.4 host gene and the *lin-4* locus (enlarged) (not to scale). Host gene exons are shown as rectangular boxes. Approximate positions of qPCR primer pairs (i-iv) are shown as small black arrows. Blue arrows indicate the direction and approximate start sites of transcription [9]. LCE = *lin-4-*complementary element. (B) qPCR analysis of primary *lin-4* (primer pair iv), F59G1.4a (primer pair i), F59G1.4b (primer pair v), F59G1.4 intron 9 (primer pair ii), and F59G1.12 antisense RNA (primer pair iii). All expression levels are relative to act-1 and normalized to the 1hr N2 time point, and expressed as a mean ± SD of two independent experiments. (C) Representative ChIP-qPCR of RNAPII occupancy across intron 9 of F59G1.4, in synchronized L1’s at 12h post-embryonic development. Error bars represent SD of technical triplicates. The experiment was performed three times with similar results.

The steady-state level of mature miRNAs can be influenced by numerous regulatory mechanisms beyond transcriptional control, including RNA degradation pathways and the processing of the primary and precursor miRNAs [18]. Thus, it was possible that the endogenous LCE did indeed function as a transcriptional regulatory element, but that its effect on *lin-4* expression was masked by post-transcriptional regulatory mechanisms. The *lin-4* gene resides in an intronic region of the F59G1.4 host gene, a poorly characterized gene expressed at relatively low and invariant levels throughout *C*. *elegans* development. *lin-4* is located in the 9^th^ intron of the F59G1.4a isoform, ~300bp downstream of an antisense transcript (F59G1.12), and ~200bp upstream of an exon and alternative transcriptional start site of its host gene (F59G1.4b) (Fig 3A). We profiled the temporal expression profile of F59G1.4a, F59G1.4b, F59G1.12, host intron 9, and pri-*lin-4*, in wild-type and *lin-4-LCE(za25)* mutants. This analysis provided a unique overview of the relationship between host gene, antisense RNA, and pri-miRNA expression, in a defined time frame in which the developmentally programmed upregulation of *lin-4* occurs.

First, we found that deletion of the LCE does not affect the steady-state levels of any of these transcripts, at any of the time points examined (Fig 3B, *i-v*). Second, the trend in expression changes over time was very similar between all transcripts: RNA levels begin to increase around 10h post-embryonic development, peak at 16–18h, and then decrease to approximately their original starting levels by 24h. However, the magnitude of these changes are different. Pri-*lin-4* levels show the highest fluctuation, increasing over 10-fold at peak expression (Fig 3B, *iv*). Together with the expression profile of mature *lin-4*, these data support a previously suggested, host gene-independent mechanism of *lin-4* expression.

However, our investigation of the host intron and flanking exons revealed a moderate, but previously unrecognized, contribution of host gene transcription to *lin-4* upregulation. We found the intron, and both isoforms of the F59G1.4 host gene mRNA, to be upregulated between ~1.5 and 3-fold between 10h-16h of post-embryonic development, suggesting that the activation of *lin-4* expression in late L1’s is not solely due to independent transcription, but partly involves host gene activation as well (Fig 3B, *i*, *ii*, *iv*).

A study by Bracht et al. [9] reported a ~4-fold increase at peak expression in pri-*lin-4* levels relative to the host intron, by semi-quantitative RT-PCR. Our *act-1*-normalized qPCR data reveals a ~2.5-fold increase in host gene intron levels and a ~10-fold increase in pri-*lin-4* levels (Fig 3B, *ii*, *iv*). Thus, relative to the host intron, we also find pri-*lin-4* expression to be increased ~4-fold. The remarkable consistency between our results not only adds confidence to the accuracy of our findings but is likely a reflection of the highly regulated nature of *lin-4* miRNA biogenesis.

As a final test of LCE function, we performed chromatin immunoprecipitation (ChIP)-qPCR to assay RNA polymerase II (RNAPII) recruitment to the *lin-4* locus in synchronized L1 animals. While our results in wild-type larvae confirm ModEncode RNAPII ChIP-Seq data, we found no significant changes in RNAPII occupancy between wild-type and *lin-4-LCE(za25)* mutants at any of the intronic DNA regions examined (Fig 3C). We conclude that the LCE not required for the transcriptional activity of the endogenous *lin-4* promoter.

### The temporal expression profile of F59G1.12, a *lin-4* promoter-associated antisense RNA, is not affected by the LCE

We also examined the temporal expression profile of F59G1.12, a *lin-4* promoter-associated antisense RNA, for three reasons. First, it contains the LCE sequence. Second, antisense transcripts have been implicated in both activating and repressing roles in regulating proximal gene expression [19]. Third, we wished to determine whether its expression was separately regulated from that of *lin-4*, which would suggest a potential regulatory function. However, the nearly identical temporal expression profile of the F59G1.12 antisense RNA with that of pri-*lin-4* indicates that it is likely a product of bidirectional transcription, where high levels of RNA polymerase activity in the sense direction often produces a measurable amount of antisense transcripts. The fact that F59G1.12 expression is unaffected in the *lin-4-LCE(za25)* mutant further confirms that the LCE does not influence the transcriptional activity of the endogenous *lin-4* promoter.

### *lin-4* autoactivation is not an endogenous regulatory mechanism

**Fig 4:**
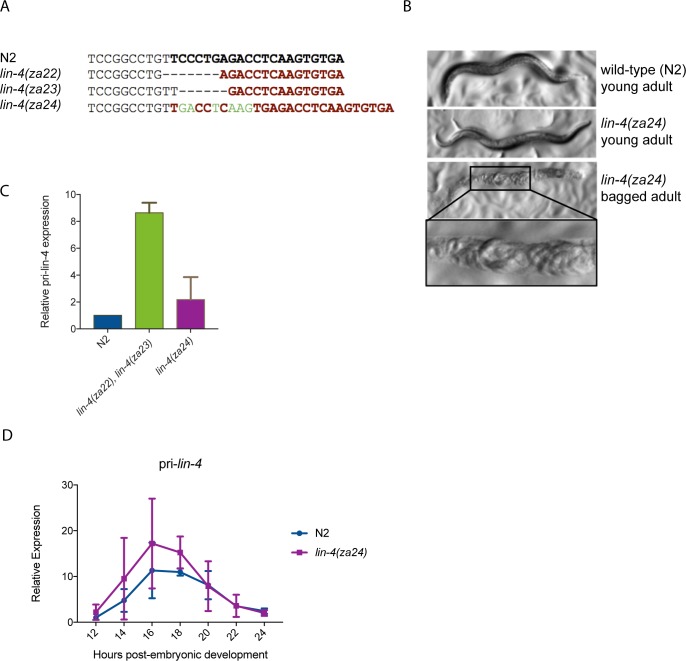
*lin-4* mutations do not impair *lin-4* promoter activity. (A) The wild-type mature *lin-4* sequence is in bold. Below are the aligned sequences of three CRISPR-generated *lin-4* mutants. Dashes indicate deleted bases; inserted bases are in green. (B) Light microscopy images of wild-type (N2) and CRISPR-generated *lin-4* mutant adults. Zoomed-in image shows hatched larva in the adult hermaphrodite, the “bagging” phenotype characteristic of *lin-4* mutants. (C) qPCR of primary *lin-4* levels in CRISPR-generated *lin-4* mutants at 12h post-embryonic development, normalized to *act-1*. The green bar indicates the average pri-*lin-4* expression of *lin-4(za22)* and *lin-4(za23)* mutants. The purple bar represents the average pri-*lin-4* expression of *lin-4(za24)* mutants (n = 2 independent replicates). Error bars represent SD. (D) qPCR time course of pri-*lin-4* expression in N2 vs. *lin-4(za24)* mutant animals. Data points are expressed as mean ± SD of two independent experiments.

We previously showed that *Plin-4*::*GFP* expression is reduced in a *lin-4(e912)* mutant background [10]. The *lin-4(e912)* mutant carries a large deletion that removes *lin-4* as well as ~5kb of upstream sequence [1], making it impossible to measure its effects on the endogenous *lin-4* promoter activity. To disrupt *lin-4* miRNA function while preserving its promoter, we used CRISPR-Cas9 to generate small indel mutations within the mature *lin-4* sequence. We generated three mutant lines, each displaying a fully penetrant *lin-4(e912)* phenotype (Fig 4A and 4B). We suspected that these mutations may also impair the processing of the pre- and pri-miRNA due to abnormal hairpin structures [20], potentially confounding our interpretation of pri-miRNA levels as a readout of *lin-4* promoter activity. Indeed, all three mutants displayed elevated primary *lin-4* expression compared to wild-type (Fig 4C). However, since the *lin-4(za24)* mutant displayed the smallest relative accumulation of primary *lin-4* transcripts compared to wild-type (a ~two-fold increase at 12h post-embryonic development), we compared the temporal expression profile of pri-*lin-4* in wild-type and *lin-4(za24)* mutants. Despite a possible mild defect in pri-*lin-4* processing, the similarity in the magnitude and timing of the peak in pri-*lin-4* levels in wild-type and *lin-4(za24)* mutants suggests *lin-4* is not required for the activity of its own endogenous promoter (Fig 4D).

## Conclusions

In summary, together with our previous work, we have identified the three core components of a miRNA regulatory module (namely *lin-4*, the LCE, and the nuclear Argonaute NRDE-3 and its co-factors), as positive regulators of *Plin-4*::*GFP* expression. However, here we show that none of these three components function as activators of the endogenous *lin-4* promoter. How might we explain this discrepancy? It is possible that *lin-4* may somehow positively regulate the translation of *Plin-4*::*GFP-*derived transcripts; indeed, examples of miRNA-mediated translational activation have been reported [21, 22]. It is also possible that the *Plin-4*::*GFP* reporter construct, which is integrated in multiple copies in an unknown genomic location in the *zaIs1* line [8], is subject to locus-specific and/or multicopy gene-specific regulatory mechanisms that are not active at the endogenous *lin-4* promoter. We do note that we have generated multiple independent strains carrying non-integrated, extragenic copies of the *Plin-4*::*GFP* reporter, which behave similarly to the *zaIs1* line, suggesting that the integration site itself is not likely the culprit. Finally, specifically regarding the importance of the LCE, it is possible that additional sequences in the endogenous *lin-4* locus act redundantly to ensure the robust upregulation of *lin-4* in early larval development.

Our data provide a prominent example of a discordant set of results between reporter-based and endogenous promoter studies. To our knowledge, few such cases have been previously described. Presumably, this is partly due to the difficulty of testing candidate regulatory sequences in endogenous contexts, at least prior to the advent of CRISPR-Cas9-mediated gene editing tools. Our work provides a cautionary tale for the interpretation of promoter elements within transgenic reporter constructs, and underscores the importance of validating the endogenous relevance of regulatory sequences identified in reporter-based systems.

## Materials and methods

### Strains and crosses

*C*. *elegans* strains were maintained as previously described. The Bristol N2 strain was used as the standard wild-type. The *zaIs1* line carries an integrated transgene consisting of the *lin-4* promoter driving GFP followed by the *unc-54* 3’ UTR (*Plin-4*::*GFP*::*unc-54)*. Additional strains used were *wIs79(ajm-1*::*GFP; scm-1*::*GFP)*, *nrde-1(gg88)*, *nrde-2(gg91)*, *nrde-3(gg66)*, and *nrde-4(gg129)*. All *nrde* mutant alleles are loss-of-function. The *zaIs1* line was crossed with N2 males, and GFP-expressing males were subsequently crossed into each of the above mutant alleles. Synchronized L1 populations were obtained by hypochlorite treatment of gravid adults followed by overnight hatching of embryos in M9 buffer. All strains were maintained at 20°C, unless otherwise specified.

### RNA interference

Bacteria from the Ahringer RNAi library carrying either the empty L4440 control vector, or dsRNAs targeting *nrde-1*, *nrde-2*, *nrde-3*, *nrde-4*, were grown to log phase, induced with 0.4mM IPTG for 4hrs, and seeded onto NGM plates containing 1mM IPTG and 50ug/ml Carbenicillin. *zaIs1(Plin-4*::*GFP)* L4 animals were picked onto fresh RNAi plates and the F1 progeny were scored for seam cell GFP expression.

### Brood size assay

Individual N2 and LCE mutant ~L3 animals were picked onto 6cm NGM plates with op50. After 7 days, the number of adult F1s were counted. In this time none of the F2 progeny became adults, ensuring an accurate count of the F1 brood size.

### Genome editing

CRISPR-Cas9-mediated gene editing was carried out as previously described [17], with minor modifications. CRISPR/Cas9 and gRNA plasmids used were Addgene p46168 and p46169. The *unc-119* gRNA sequence downstream of the *pU6* promoter in p46169 was replaced with gRNAs 5’- GTGGCACCTAACACTATTTC -3’, or 5’- CACTTGAGGTCTCAGGGAAC -3’, to target the LCE, or *lin-4*, respectively, by overlap PCR. The PCR product was cloned back into p46169 by digestion with EcoRI and HindIII. Microinjections were performed with 30–40ng/uL of guide RNA plasmid, 7ng/uL of p46169 (*Peft-3*::*Cas9)*, 50ng/uL 1kb DNA ladder (NEB), and 5ng/uL of *Pmyo-2*::*dsRED* co-injection marker. For the *lin-4-*targeting injections, individual transgenic F1’s were isolated based on *Pmyo-2*::*dsRED* expression. F2’s that displayed a *lin-4(e912)* phenotype were genotyped by Sanger sequencing across the *lin-4* miRNA locus. For the LCE-targeting injections, we again isolated individual transgenic F1’s, but since LCE disruptions were not certain to show a phenotype, we pooled ~10–15 F2’s from each transgenic F1 for sequencing across the LCE. Progeny from plates that gave a heterozygous sequence were then cloned out and further sequenced until a homozygous LCE mutant line was established.

### Microscopy

For seam cell GFP expression, V-lineage divisions, and alae production, animals were immobilized in 1mM levamisole and examined using an upright Zeiss Axioplan microscope under 40x and 63x magnification.

### Chromatin immunoprecipitation (ChIP)

Synchronized 12h L1’s were fixed in 2% formaldehyde for 30 minutes at room temperature, washed once in 100mM Tris pH 7.5, twice in M9, and frozen at -80C. Frozen samples were resuspended in ~500ul FA buffer (50mM HEPES/KOH pH 7.5, 1mM EDTA, 1% Triton X-100, 0.1% sodium deoxycholate, 150mM NaCl), and sonicated using a Branson digital sonifier at 4oC on ice, at 30% amplitude, 5 sec on, 45 sec off, for 8 cycles. Samples were spun at 13000g for 15 minutes at 4oC, and protein concentration was determined by Bradford assay. Immunoprecipitations were performed with ~0.5mg of protein, in 500uL total volume in FA buffer in ~1% sarkosyl and 1x protease inhibitors (Roche), using 10uL of the GW816 mouse monoclonal RNA polymerase II antibody (Santa Cruz). Immunocomplexes were rotated overnight at 4oC. The next day, 50uL of protein A sepharose beads were added, and rotated at 4oC for 2.5hrs. The beads were washed twice in FA buffer, once in FA buffer with 1M NaCl, once in FA buffer with 0.5M NaCl, once in TEL buffer (0.25M LiCl, 1% NP-40, 1% sodium deoxycholate, 1mM EDTA, 10mM Tris-HCl pH 8), and twice in TE. Immunocomplexes were eluted in 150uL 1% SDS in TE with 250mM NaCl at 65oC for 15mins; this step was performed twice and the eluates were combined. 2uL of 10mg/mL proteinase K was added and samples incubated at 55oC for 1–2hrs and then 65oC overnight. DNA was purified using a PCR purification kit (Qiagen).

### RNA extraction, cDNA synthesis and quantitative PCR (qPCR)

*C*. *elegans* samples were collected in M9, pelleted by centrifugation, and flash frozen in liquid nitrogen. RNA was isolated using a standard Trizol (Invitrogen) protocol, or with Direct-zol Miniprep Plus spin columns (Zymo Research). The RNA was treated with 0.5uL of Turbo DNase (Ambion) for 20 minutes at 37°C, and then with DNase Inactivation Reagent (Ambion). cDNA synthesis was performed using Superscript III (Invitrogen) and random hexamers. Quantitative real-time PCR was performed using SYBR Green (Applied Biosystems) on a Roche LightCycler 480.

For qPCR analyses of transcripts around the endogenous *lin-4* locus, animals were bleached and embryos were allowed to hatch overnig ht in M9. The following day, the time at which synchronized L1’s were plated onto op50 plates was considered 0hr and the start of the time course. Total RNA was collected, DNase-treated, and reverse transcribed as described above, with two exceptions: (1) The F59G1.12 antisense transcript was reverse transcribed using a single, gene-specific primer F59G1.12 RT1: 5’-CGTCTCTGTGGCACCTAACA-3’; (2) U18 and mature *lin-4* RNA were reverse transcribed with Taqman probes RT00176 and RT00258, respectively, according to the manufacturer’s protocol. All qPCR primer efficiencies were tested and only those with an efficiency between 85–115% were used. All gene expression was normalized to *act-1* mRNA, with the exception of mature *lin-4* miRNA, which was normalized to U18. Fold changes were calculated using the ΔΔCt method. Primers used in this study are shown in Table 2.

**Table 2:** Primers used in this study.

Primers for SYBR Green-based qPCR for Gene Expression		
Gene / Genomic region	F- Primer Sequence (5' to 3')	R- Primer Sequence (5' to 3')
act-1	GTTCACCGCAAGTGCTTCTAAATG	GCAAATGAGTGAAAGGACAATAAGG
pri-lin-4	CTAGACAATTTCTAGAGTTTTGGTTGGT	GGAACTAGCTCCCAGTGTGAAAA
F59G1.4a	CGACTTGCTGATTCTTTTGC	ACCGGATACGAATAGCCTCA
F59G1.4b	GAAAAGCACCATCTCTGCTG	TTTTCTCTGACGGTGGTGAT
F59G1.4 Intron 9	AGAGCATTGCCTTTTCCCTA	TCCCTCAAATGGGTACATGA
F59G1.12	TAGCTGAAGGAGGAGGATCG	GTGTCCCTCCGTGCTCTG
Primers for RNA Pol II ChIP-qPCR (distance upstream of mature lin-4 in kb)		
Intron 9 (-3.475)	ACTCCGTCGTAGTAACCCATAAC	TTGGCTCTCTGTAATCCAACAATTCAA
Intron 9 (-1.98)	AGAGCATTGCCTTTTCCCTA	TCCCTCAAATGGGTACATGA
Intron 9 (-0.653)	CCTTTCCCACCCATTATGTC	CGGCTGTTTAGGGAGAAGAA
Intron 9 (-0.2)	GACCGAATGACCCAGTCTCT	AGTCCGACCGATTGTGGTAG
Intron 9 (0)	CTAGACAATTTCTAGAGTTTTGGTTGGT	GGAACTAGCTCCCAGTGTGAAAA
